# Preliminary Investigation of Shift, a Novel Smartphone App to Support Junior Doctors’ Mental Health and Well-being: Examination of Symptom Progression, Usability, and Acceptability After 1 Month of Use

**DOI:** 10.2196/38497

**Published:** 2022-09-21

**Authors:** Samineh Sanatkar, Isabelle Counson, Andrew Mackinnon, Alexandra Bartholomew, Nick Glozier, Samuel Harvey

**Affiliations:** 1 Black Dog Institute Randwick Australia; 2 School of Psychiatry Faculty of Medicine University of New South Wales Sydney Kensington Australia; 3 Central Clinical School Faculty of Medicine and Health University of Sydney Camperdown Australia

**Keywords:** digital mental health, mobile health apps, mHealth apps, help seeking, junior doctors, depression, mobile phone

## Abstract

**Background:**

*Shift* is a novel smartphone app for providing a digital-first mental health resource to junior doctors. It contains psychoeducational material, cognitive behavioral modules, guided mediations, information on common work stressors, and a section on help-seeking options for psychological problems through workplace and private avenues.

**Objective:**

This study aimed to conduct a preliminary investigation of the use and potential effectiveness of *Shift* on depressive and anxiety symptoms (primary outcomes) and work and social functioning, COVID-19 safety concerns, and help seeking (secondary outcomes). This study also sought feedback on whether *Shift* was seen as an acceptable tool.

**Methods:**

Junior doctors in New South Wales, Australia, were approached through promotional activities from the Ministry of Health, specialist medical colleges, and social media advertisements between June and August 2020. Consenting participants provided web-based baseline data, used the *Shift* app for 30 days, and were asked to complete a poststudy web-based questionnaire. Outcomes were analyzed under the intention-to-treat principle.

**Results:**

A total of 222 (n=156 female, 70.3%; mean age 29.2, SD 4.61 years) junior doctors provided full baseline data. Of these, 89.2% (198/222) downloaded the app, logged into the app approximately 6 times (mean 5.68, SD 7.51), completed 4 in-app activities (mean 3.77, SD 4.36), and spent a total of 1 hour on in-app activities (mean 52:23, SD 6:00:18) over 30 days. Postintervention and app use data were provided by 24.3% (54/222) of participants. Depressive and anxiety symptoms significantly decreased between the pre- and postassessment points as expected; however, physicians’ COVID-19 safety concerns significantly increased. Work and social functioning, COVID-19 concerns for family and friends, and help seeking did not change significantly. There was no significant relationship between symptom changes and app use (number of log-ins, days between first and last log-in, and total activity time). Most poststudy completers (31/54, 57%) rated *Shift* highly or very highly.

**Conclusions:**

Despite high levels of nonresponse to the poststudy assessment and increases in COVID-19 safety concerns, junior doctors who used the app reported some improvements in depression and anxiety, which warrant further exploration in a robust manner.

## Introduction

### Background

Psychological distress and mental health disorders such as major depression are prevalent in junior doctors [1,2]. A recent review and meta-analysis identified high work demands, patient care concerns, and a poor work environment as the most important workplace risk factors associated with the decline of junior doctors’ mental health [1]. Individual risk factors such as low perceived mental and physical well-being and poor self-efficacy were also identified, although they were generally less robust predictors of distress [1]. Poor mental health can negatively affect junior doctors’ prospects at a time that may be critical for establishing their personal life and career goals. For example, poor perceived mental and physical health among doctors is associated with lower job satisfaction and intentions to leave medicine altogether [3]. Particularly among junior doctors, job burnout is further associated with decreased professionalism and lower patient safety [4]. Thus, when severe distress impacts workplace functioning, the quality of care provided to patients and early-career physicians’ long-term job prospects may be negatively impacted [5,6].

National survey data indicated that 21% of surveyed Australian doctors had been diagnosed with major depression, and 9% had received an anxiety disorder diagnosis in their lifetime, with junior doctors reporting particularly high psychological distress at the time of survey completion [7]. A recent review and meta-analysis suggested that psychological interventions aimed at reducing common mental health disorders in physicians lessen disease burden [8]. Although this evidence is promising, the authors also noted that the delivery and acquisition of cognitive behavioral and mindfulness techniques requires doctors to set aside time and resources to pursue such therapy [8]. This could constitute a substantial barrier to entering treatment. The authors suggested that it is therefore advised to explore the option of delivering therapeutic components on the web or via smartphone apps to increase accessibility [8]. To our knowledge, no attempt has been made to date to convey the principles of cognitive behavioral therapy (CBT) and mindfulness to populations via a smartphone app.

Australian junior doctors seek psychological treatment for depression at lower rates than senior doctors [9]. In addition to issues related to mental health stigma in the medical profession [10,11], one practical reason for this may be a gap in the knowledge on how to seek such support. A recent large-scale Australian survey indicated that one-quarter of surveyed junior doctors (1929/7715, 25%) did not know or were unsure of how to seek help for their physical and mental health concerns [12]. Although some mental health problems remit over time [13], prognosis is generally best if individuals seek treatment early on [14,15]. Untreated persistence of mental health problems over prolonged periods reduces recovery rates, even when therapy is eventually sought [15]. Thus, facilitating access to professional support early on may be an important factor in increasing the success rate of interventions aimed at improving junior doctors’ mental health.

Several prominent mental health crises in junior physician populations have been documented in the media in recent years [16,17]. In the Australian context, a cluster of 3 junior doctors’ suicides in New South Wales gained widespread public interest, and calls for reforms were made from inside and outside the medical profession [16,18]. As a response to these calls and after consultation with relevant parties, including junior doctors, the New South Wales Ministry of Health issued a 10-point Junior Medical Officer Wellbeing and Support Plan [19]. One of the 10 initiatives was the development and implementation of the *Shift* smartphone app. *Shift* was developed to provide a discreet resource for junior doctors in New South Wales to access mental health–related content and information on how to seek mental health care.

*Shift* digitally delivers therapeutic components adapted from CBT, acceptance and commitment therapy, mindfulness, and psychoeducational material shown to be useful in physician and health care worker populations [8,20,21] with the primary aim of reducing the symptoms of depression and anxiety. The current version of *Shift* is a result of an iterative development process involving junior doctors in qualitative interviews, user experience workshops, and pilot testing of the prototype version of the app. The full development and testing process is outlined in a previous publication [22]. The COVID-19 outbreak coincided with the recruitment phase of this study. To respond to the new situation, 2 clinical psychologists and a psychiatrist at the Black Dog Institute (SH) helped develop novel contents on health anxiety and COVID-19 concerns, the latter of which was prominently placed within the app.

### Objective

This study sought preliminary evidence of the effectiveness of the *Shift* app among junior doctors. This study was planned as a 2-arm waitlist-controlled randomized controlled trial, but as a response to the COVID-19 pandemic and to ensure that all junior doctors interested in taking part in this investigation could download the app without delay, this study was implemented as an uncontrolled quasi-experimental pre-post intervention evaluation with the primary aim of assessing depressive and anxiety symptom levels over the course of 1 month of using the app. Relatedly, the study examined whether potential improvements in depressive symptoms would help restore functioning across work and private life domains and whether COVID-19 pandemic concerns remitted over the course of the study. Where significant improvements were observed, we aimed at investigating whether there was a relationship between app use and symptom reduction. Another aim was to encourage help seeking for mental health problems by way of providing in-app information on the various workplace and private avenues of available support and by alleviating potential concerns around mandatory reporting guidelines.

## Methods

### Study Design

**Figure 1:**
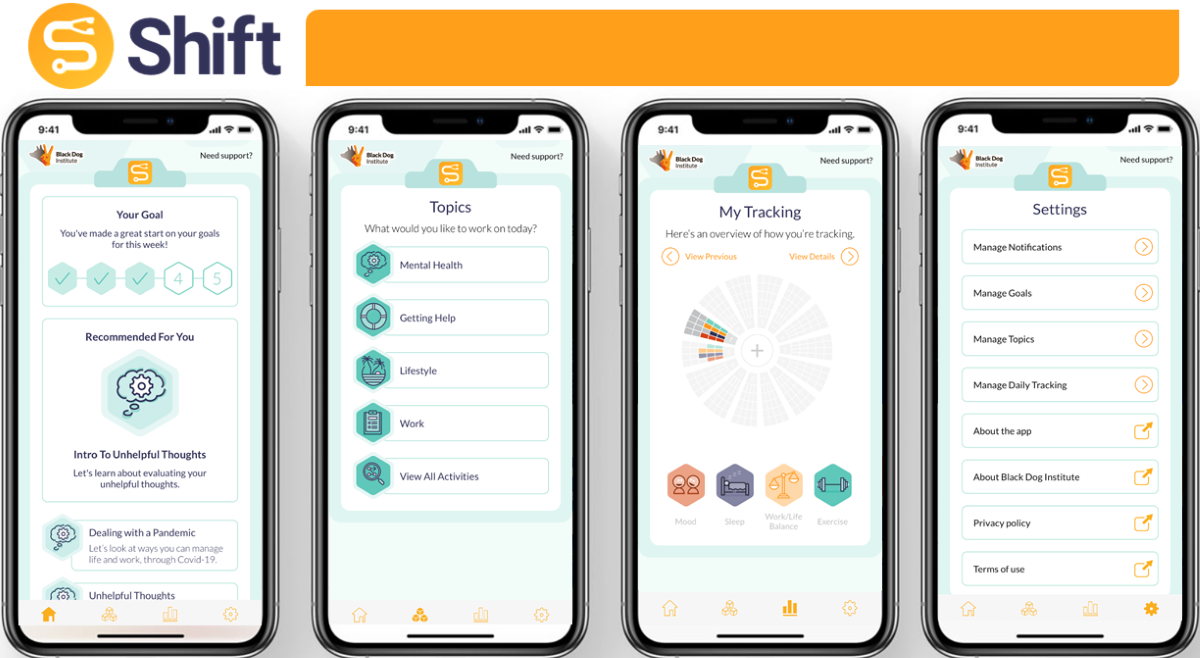
Examples of *Shift* app screen designs.

We conducted an uncontrolled quasi-experimental pre-post intervention to examine the trajectories of depression and anxiety symptoms (primary outcomes) and COVID-19 pandemic concerns, work and social functioning, and help-seeking tendencies (secondary outcomes). This information was provided via web-based questionnaires. *Shift* app use data were automatically collected in the 1-month period between the participants’ download of *Shift* and the poststudy survey assessment. An overview of the app layout design can be seen in Figure 1. At the end of the study period, we reassessed the participants’ symptom scores and asked the participants to provide app usability and acceptability ratings using web-based questionnaires.

Mixed model repeated measures ANOVA was used to investigate pre- and postsymptom change in the primary and secondary outcome variables. This method was used in preference to paired sample *t* tests to retain all observations. It also makes less stringent assumptions regarding missingness mechanisms. We further explored whether app use was indicative of significant symptom trajectories. The relation between app use and poststudy symptom levels was examined using linear regression analyses, whereby postintervention symptom scores were the dependent variable and app use variables were entered as independent variables along with baseline symptom scores as a covariate.

### Participants

Between June and August 2020, junior medical officers practicing in New South Wales, Australia, were invited to join the *Shift* app research study. Recruitment took place through three different avenues: (1) the New South Wales Ministry of Health promotional efforts through an official media release and at relevant meetings or forums, (2) liaison with medical colleges and other organizations to disseminate study recruitment material, and (3) through a social media campaign via Facebook and on the Black Dog Institute website. Disseminated recruitment materials contained study details (eg, study components, app contents, and study duration), ethical information (eg, voluntary participation and data handling), and a link to a purpose-designed study website that provided the full Participant Information Statement and Consent Form as a downloadable file.

Eligibility criteria were currently being employed as an intern, a resident, a registrar, or a junior career medical officer in New South Wales and owning an internet-enabled smartphone with an iOS or Android operating system. After consenting and confirming eligibility through screening, the participants completed a web-based questionnaire containing demographic information and mental health–related questions. Subsequently, the participants were given instructions on how to download the *Shift* app from the App Store or Google Play Store. The onboarding of the *Shift* app was completed by the participants, which included prompts to set preferences with regard to whether to allow app notifications, and if yes, at which intervals, and to set weekly goals for app activity completion. These settings could be modified within the app at any time. After 30 days, the participants were asked to complete a postintervention web-based assessment questionnaire. Items reassessed the participants’ mental health status and asked for *Shift* app feedback. Measurement tools are provided in the subsequent sections.

### Measures

#### Depression

The participants completed the 9-item Patient Health Questionnaire [23], which assesses major depression symptom severity. The participants responded to items such as “feeling down, depressed, or hopeless” on a 4-point Likert-type scale ranging from 0 (*not at all*) to 3 (*nearly every day*). Total scores range from 0 to 27, with scores of ≥10 being indicative of possible major depressive disorder. The measure has been shown to be reliable and valid and has been used in physician samples, including training physicians [23,24].

#### Anxiety

General anxiety symptom levels were measured using the 7-item Generalized Anxiety Disorder scale [25]. The participants indicated their agreement with items such as “feeling nervous, anxious or on edge” on a Likert-type scale ranging from 0 (*not at all*) to 3 (*nearly every day*). Total scores on the 7-item Generalized Anxiety Disorder scale range from 0 to 21, with scores ≥10 being indicative of symptoms consistent with general anxiety disorder. The measure has been found to have good psychometric properties and has been used in physician samples, including during the COVID-19 pandemic [25-27].

#### Work and Social Functioning

Functioning in social and work arenas was measured using the 5-item Work and Social Adjustment Scale [28]. The participants responded to items such as “Because of my problems, my ability to work is impaired” on an 8-point Likert-type scale anchored at 0 (*not at all*) and 8 (*very severely*). Scores on the Work and Social Adjustment Scale range from 0 to 40, with higher scores indicating reductions in work and social functioning. Specifically, scores between 10 and 20 are indicative of significant functional impairment, and scores >20 indicate functional impairment on clinical symptom levels.

#### COVID-19 Safety Concerns

The participants’ safety concerns regarding the possible contraction of COVID-19 were assessed using 2 items developed for the study. The participants indicated their concerns for their own safety (“How concerned or worried are you that you, personally, will catch COVID-19?”) and the safety of their family and friends (“How concerned or worried are you that your family members or friends will catch COVID-19?”) on a 5-point Likert-type scale ranging from 1 (*not at all*) to 5 (*extremely*).

#### Help Seeking

To assess the participants’ willingness to seek help for mental health concerns, we modified the first item of the General Help-Seeking Questionnaire [29]. The original item “If you were having a personal or emotional problem, how likely is it that you would seek help from the following people?” was amended to “If you were having a personal or emotional problem, how likely is it that you would seek help from a mental health professional?” The participants indicated their responses on a 7-point Likert-type scale ranging from 1 (*extremely unlikely*) to 7 (*extremely likely*). To assess recent actual help-seeking behaviors for mental health concerns, we created the following single-item indicator: “Did you seek any help for a personal or emotional problem from a mental health professional in the past month?” The participants responded to this item using a “yes” or “no” response option.

#### App Acceptability

The poststudy questionnaire included several *Shift* app acceptability and usability items. Acceptability items were as follows: “What is your overall rating of the app?” “I would use this app again in the future.” “I would recommend this app to other Doctors in Training.” The participants indicated their overall rating of the app on a 5-point Likert-type scale ranging from 1 (*very poor*) to 5 (*very high*) and stated their agreement with the future app use and recommendation items on a 5-point Likert-type response scale anchored at 1 (*strongly disagree*) and 5 (*strongly agree*). For a brief indication of the perceived usability of the *Shift* app, the participants were asked to respond to a modified single-item indicator adopted from the System Usability Scale [30], termed “I thought the app was easy to use.” The participants indicated their level of agreement with this item on a 5-point Likert-type response scale anchored at 1 (*strongly disagree*) and 5 (*strongly agree*). As a part of the app onboarding process, the participants set their own app use goals (the app recommended the completion of between 3 and 5 activities per week). The participants were asked whether they reached their own app use goals: “On average, did you hit your weekly challenge goal or not?” to which the participants indicated 1 (*hit goal*) or 0 (*missed goal*).

#### App Use Variables

*Shift* app use measurement included the total number of log-ins, days between the first and last log-in (maximum 30 days), and the number of activities started and completed. Examples for cognitive behavioral activities were “unhelpful thoughts” and “evaluating thoughts.” “Sleep health” and “adjusting to shift work” were examples for work and lifestyle activities. A full list of activities and how they were organized within the app can be found in Multimedia Appendix 1. Total activity time and times spent on each of the cognitive behavioral, work and lifestyle, mindfulness, psychoeducation, value-based actions, and “get help” sections were also recorded, although it was not possible to distinguish between the times when users engaged with the app contents and the times when the app was open but not attended to.

### Ethics Approval

The study was prospectively registered with the Australian New Zealand Clinical Trials Registry under the trial number ACTRN12620000571976. Ethics approval for this study was obtained through University of New South Wales Sydney Human Research Ethics Committee (protocol number HC200212).

## Results

### Sample Characteristics

#### Participant Flow

**Figure 2:**
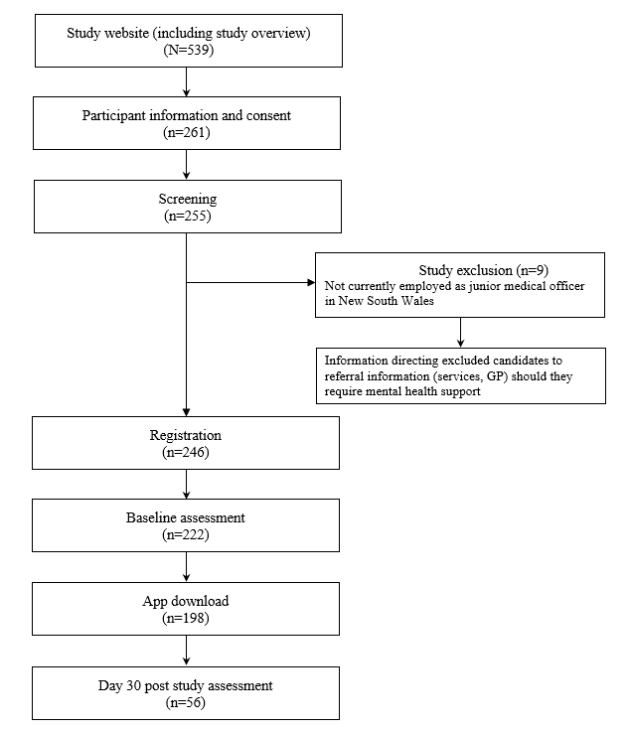
Flowchart illustrating how participants progressed through the Shift study. GP: general practitioner.

Figure 2 illustrates the participant flow through the *Shift* study. Of the 539 doctors who visited the study website, 261 (48.4%) consented to participate and 255 (47.3%) completed the study screen. Of these 255 candidates, 9 (3.5%) were ineligible to register for the study because they were not currently employed as junior doctors in New South Wales, and 24 (9.4%) candidates did not complete the baseline assessment, resulting in a final sample size of 222 study participants. Although all but 1.8% (4/222) of participants went on to download the *Shift* app as directed, only 68.9% (153/222) accessed the *Shift* app more than once. Attrition in the poststudy assessment was high, with 25.2% (56/222) of the participants, that is, a quarter of baseline completers, returning the full poststudy assessment after 1 month. A total of 4% (2/56) of participants returned the baseline and poststudy assessments but did not download the app. Consequently, complete data were available for 24.3% (54/222) of the participants.

#### Baseline Sample Composition

The participants were predominantly female (156/222, 70.3%) and had a mean age of 29.2 (SD 4.61) years. Most participants were employed as registrars (85/222, 38.3%), interns (55/222, 24.8%), or residents (50/222, 22.5%) in metropolitan areas (137/222, 61.7%). Symptoms of poor mental health were noted among some participants; 23.9% (53/222) reported depressive symptoms consistent with possible major depression, 14.4% (32/222) reported symptoms indicative of general anxiety disorder, and 9.5% (21/222) indicated clinically significant levels of functional impairment. In the month preceding the baseline assessment, 19.5% (43/222) had sought professional help for mental health concerns, and 32.9% (73/222) of doctors indicated having had contact with a patient with observed or covert COVID-19.

Table 1 shows the baseline characteristics of participants who completed only the baseline assessment (166/222, 74.8%) and those who completed the baseline and also poststudy assessments (56/222, 25.2%). Comparisons of demographic (age and gender), mental health, functioning, COVID-19 concerns, and help-seeking information at baseline did not suggest any systematic differences between the study completers and those who did not complete the poststudy assessment (all *P*>.05; Table 1). The standardized mean differences between those who completed both the pre- and postassessments and those who were lost to follow-up were small, with corrected effect size values (Hedges *g*) ranging from 0.08 for personal COVID-19 safety concerns to 0.29 for work and social functioning.

**Table 1:** Sample characteristics at baseline (N=222), comparing participants who completed the prestudy assessment (n=166) and those who completed pre- and poststudy assessments (n=56).

Characteristics		Prestudy assessment only		Pre- and poststudy assessment				*F/χ*^2^ (*df*)	*P* value			Hedges g/Cohen *w*		
**Demographics**														
	Age (years), mean (SD)		28.93 (4.61)		29.8 (4.6)	1.49 (1)				.22			0.19	
	Female, n (%)		118 (71.1)		38 (67.9)	1.89 (2)				.39			0.09	
**Level of training, n (%)**							4.67 (4)				.32			0.10
	Registrar		58 (34.9)		27 (48.2)									
	Intern		45 (27.1)		10 (17.9)									
	Resident		40 (24.1)		10 (17.9)									
	Senior resident		22 (13.3)		9 (16.1)									
	Junior career medical officer		1 (0.6)		0 (0)									
**Specialty^a^, n (%)**							26.10 (21)				.20			0.11
	General medicine		11 (6.6)		3 (5.4)									
	Psychiatry		10 (6.0)		2 (3.6)									
	Emergency medicine		8 (4.8)		5 (8.9)									
	Pediatric and child health		7 (4.2)		5 (8.9)									
**Work location, n (%)**							1.54 (2)				.46			0.08
	Metropolitan		99 (59.6)		38 (67.9)									
	Regional		53 (31.9)		13 (23.2)									
	Rural		14 (8.4)		5 (8.9)									
**Mental health, mean (SD)**														
	Depression		6.99 (4.12)		6.39 (5.03)	0.78 (1)				.38			0.14	
	Anxiety		5.45 (3.94)		5.07 (4.39)	0.36 (1)				.55			0.09	
	Work and social adjustment		10.92 (7.32)		8.84 (6.77)	3.49 (1)				.06			0.29	
**COVID-19 pandemic concerns, mean (SD)**														
	Self		1.90 (0.77)		1.84 (0.68)	0.26 (1)				.61			0.08	
	Family and friends		2.57 (1.01)		2.48 (0.95)	0.30 (1)				.59			0.09	
**Help seeking**														
	Willingness to seek help, mean (SD)		4.14 (1.41)		3.91 (1.24)	1.15 (1)				.28			0.17	
	Sought help in past month, n (%)		33 (19.9)		10 (17.9)	0.11 (1)				.74			0.02	

^a^Only the most common specialty areas are listed out of a total of 22.

### Primary Outcomes

#### Depression Symptom Changes

As shown in Table 2, depression symptom scores significantly decreased between the pre- (estimated marginal mean [EMM] 6.84, SE 0.29) and poststudy (EMM 5.24, SE 0.38) assessments, with t_74.89_=4.34 and *P*<.001. Glass delta effect sizes for depression symptoms changes (using the SD at baseline) were small to medium (Δ=0.37).

**Table 2:** Linear mixed effect models to examine changes in responses between pre- and poststudy assessments.

Variable			Prestudy EMM^a^ (SE)		Poststudy EMM (SE)		*t* test (*df*)		*P* value		Δ^b^
**Depression**			6.84 (0.29)		.24 (0.38)						
	Intercept					13.94 (80.25)		<.001		N/A^c^	
	Time					4.34 (74.89)		<.001		.37	
**Anxiety**			5.35 (0.27)		4.44 (0.42)						
	Intercept					10.52 (70.65)		<.001		N/A	
	Time					2.20 (67.16)		.03		.22	
**Functioning**			10.40 (0.49)		9.77 (0.78)						
	Intercept					12.51 (71.16)		<.001		N/A	
	Time					0.84 (62.42)		.41		.09	
**COVID-19 pandemic concerns, self**			1.88 (0.05)		2.05 (0.09)						
	Intercept					25.47 (70.33)		<.001		N/A	
	Time					−2.16 (64.77)		.04		.23	
**COVID-19 pandemic concerns, family**			2.55 (0.07)		2.60 (0.10)						
	Intercept					25.66 (71.21)		<.001		N/A	
	Time					−0.60 (66.10)		.55		.06	
**Help-seeking willingness**			4.08 (0.09)		4.03 (0.15)						
	Intercept					26.57 (72.61)		<.001		N/A	
	Time					0.35 (60.77)		.73		.04	
**Help seeking in the past month**			.19 (0.03)		.13 (0.04)						
	Intercept					3.10 (61.17)		.003		N/A	
	Time					1.50 (72.82)		.14		.17	

^a^EMM: estimated marginal mean.

^b^Δ: Glass delta effect size.

^c^N/A: not applicable.

#### Anxiety Symptom Changes

Results from linear mixed effects analysis (Table 2) indicated that anxiety symptoms significantly decreased between the pre- (EMM 5.35, SE 0.27) and poststudy (EMM 4.44, SE 0.42) assessments, with t_67.16_=2.20 and *P*=.032. Glass delta effect sizes for anxiety symptom changes were small (Δ=0.22).

### Secondary Outcomes

#### Functioning, COVID-19 Pandemic Concerns, and Help-Seeking Changes

Table 2 further summarizes the secondary outcomes pertaining to expected improvements in work and social functioning, personal safety concerns, concerns regarding family and friends contracting COVID-19, willingness to seek help, and recent help-seeking behaviors. Although work and social functioning scores improved as expected, changes between the pre- and poststudy assessment points were not significant (*P*>.05). Pandemic-related concerns toward participants’ own safety significantly increased for poststudy completers (EMM 2.05, SE 0.09) compared with baseline reports (EMM 1.88, SE 0.05; t_64.77_=−2.16; *P*=.04; Δ**=**0.23). Concerns for family members and friends as well as help-seeking intentions largely remained unchanged and were not significant.

#### App Use

Over the 30-day study period, the participants logged in an average of 6 times (mean 5.68, SD 7.51) and used the app over a period of approximately 2 weeks (mean 14.50, SD 12.78). On average, the participants started approximately 5 activities (mean 5.44, SD 5.94) during this time and completed 4 of them (mean 3.77, SD 4.36). Overall, the participants spent just under an hour on the activities presented within the app (mean 52:23, SD 6:00:18). CBT module use constituted the most used component with an average time spent on CBT activities of about 12 minutes (mean 12:05, SD 1:18:42), followed by about 4 minutes on work and lifestyle activities (mean 03:55, SD 10:39) and 3 minutes on mindfulness (mean 03:05, SD 11:54). On average, the participants spent the least time viewing psychoeducation (mean 00:22, SD 01:59), values (mean 00:11, SD 01:12) and get help (mean 00:04, SD 00:34) activities. Owing to the large range of times recorded, we assumed that some participants had their app open without actively engaging with the contents, prompting us to remove outliers. Although outliers >3 SDs from the mean were removed, the variability of app use data remained large.

App use behavior differed substantially between study completers and those lost to follow-up, as shown in Table 3. App use means of study completers for log-in, days between first and last log-in, and app activity use were approximately 1 SD above those who did not return the poststudy assessment (Hedges *g* ≥1.0), and the overall time spent on activities was over half an SD greater among study completers (Hedges *g*=0.61). Although there seemed to be no significant differences with regard to baseline characteristics, participants who completed the poststudy questionnaire were more involved in the month-long app use component of this research than those who did not complete the poststudy questionnaire.

**Table 3:** Participants’ 30-day *Shift* app use and poststudy app acceptability ratings, comparing participants who downloaded *Shift* and completed only the prestudy assessment (n=144) and those who downloaded *Shift* and completed pre- and poststudy assessments (n=54)^a^.

Variable	Prestudy assessment only, mean (SD)	Pre- and poststudy assessment, mean (SD)	*F* (*df*)	*P* value	Hedges *g*
Log-ins	3.56 (3.10)	11.45 (11.83)	54.50 (1,195)	<.001	1.18
Days between first and last log-in	11.26 (11.82)	23.09 (11.16)	40.46 (1,195)	<.001	1.02
Activities started	4.01 (4.91)	9.45 (6.74)	37.62 (1,196)	<.001	1.00
Activities completed	2.68 (3.54)	6.88 (4.97)	41.74 (1,196)	<.001	1.06
Total activity time^b^	12:23 (27:42)	01:08:13 (02:51:22)	14.30 (1,195)	<.001	0.61
Time spent on CBT^c^	05:35 (20:09)	29:17 (02:26:18)	3.60 (1,196)	.06	0.30
Time spent on work and lifestyle	02:22 (06:39)	08:13 (16:52)	12.14 (1,194)	.01	0.56
Time spent on mindfulness	02:24 (12:50)	04:57 (08:43)	1.80 (1,196)	.18	0.21
Time spent on psychoeducation	00:12 (01:07)	00:48 (03:21)	3.48 (1,193)	.06	0.30
Time spent on values	00:09 (01:10)	00:17 (01:18)	0.37 (1,189)	.55	0.11
Time spent on get help	00:03 (00:34)	00:08 (00:35)	0.76 (1,196)	.38	0.15

^a^Outliers >3 SDs from the mean were removed from use data.

^b^Activity time is given in minutes and seconds (00:00) or hours, minutes, and seconds (00:00:00).

^c^CBT: cognitive behavioral therapy.

#### App Use Mental Health Trajectory Relation

Given the sample size, 1 use predictor was examined at a time. Where present, outliers 3 SDs from the mean were removed for these analyses because we assumed that very long use times indicated that the app was open but not actively used. Very high correlations among the number of activities started, activities completed, and time spent on activities (*r* ranged between 0.83 and 0.96) indicated that these variables measured a singular construct (ie, activity engagement). To avoid redundancy in use variables, we only entered the time spent on activities, number of log-ins, and days between participants’ first and last log-in. As indicated in Table 4, none of the app use measures predicted depression and anxiety outcomes or COVID-19 pandemic safety concerns (*P*>.05). Although the effects for depression and anxiety were in the expected direction (eg, each new log-in predicted about 0.7 of a point reduction on the 9-item Patient Health Questionnaire), no significant relation between app use and symptom change could be detected in the present sample with the inclusion of log-in and activity use count data.

**Table 4:** Linear regression analyses of the relation between app use variables and changes in depression and anxiety symptoms and junior doctors’ COVID-19 safety concerns, with baseline values entered as covariates (N=54).

Variable			Constant, b (SE-b)		Baseline, b (SE-b)		b		SE-b		β		*R*^2^ value		Δ *R*^2^ value		Δ *F* (*df*)		*P* value
**Depression**																			
	Log-ins	2.14 (1.13)		0.52 (0.07)		−0.65		1.00		−0.06		0.52		0.00		0.42 (1,50)		.52	
	Days between first and last log-in	1.64 (1.14)		0.53 (0.07)		−0.20		0.82		−0.02		0.51		0.00		0.06 (1,51)		.81	
	Total activity time (min)	11.89 (0.86)		0.53 (0.07)		−0.40		0.52		−0.08		0.52		0.01		0.59 (1,51)		.45	
**Anxiety**																			
	Log-ins	1.80 (1.30)		0.60 (0.10)		−0.44		1.15		−0.04		0.43		0.00		0.15 (1,50)		.70	
	Days between first to last log-in	2.08 (1.24)		0.61 (0.10)		−0.61		0.93		−0.07		0.44		0.01		0.43 (1,51)		.51	
	Total activity time (min)	11.92 (0.97)		0.60 (0.10)		−0.43		0.59		−0.08		0.44		0.01		0.54 (1,51)		.47	
**COVID-19 pandemic concerns, self**																			
	Log-ins	11.00 (0.28)		0.48 (0.12)		.15		0.22		.08		0.27		0.01		0.44 (1,50)		.51	
	Days between first to last log-in	0.92 (0.29)		0.47 (0.12)		.18		0.18		.49		0.27		0.01		0.99 (1,51)		.32	
	Total activity time (minutes)	11.02 (0.25)		0.47 (0.12)		.11		0.12		.12		0.27		0.01		0.92 (1,51)		.34	

#### App Acceptability Ratings

The participants completed app acceptability items as part of the poststudy survey assessment. All average ratings were above the midpoint of the response scales. Endorsement of the ease-of-use item was good (mean 4.09, SD 0.73) as well as the participants’ willingness to use the app again in the future (mean 3.67, SD 0.97) and their willingness to recommend the app to colleagues (mean 3.70, SD 0.92). The overall rating of the app was satisfactory (mean 3.67, SD 0.75), with 57% (31/54) of the participants stating that their overall rating of the app was high or very high. Only 32% (17/54) of the participants reported having met their app use goals at the end of the study period.

## Discussion

### Principal Findings

There has been increasing international concern regarding the mental health and well-being of junior doctors [31]. Although there is emerging evidence that junior medical staff can be taught skills that will improve their mental health and reduce their risk of future mental health problems, the practical implementation of such training has proven to be a major barrier. This study represents the first ever evaluation of a smartphone app designed to meet the mental health and well-being needs of junior doctors. If shown to be effective, this type of app could be used widely at minimal cost and with minimal disruption. The results of this initial evaluation of the *Shift* app were mixed. There was evidence of a significant reduction in depression and anxiety symptoms. However, doctors’ COVID-19 concerns regarding their personal safety significantly increased. Furthermore, there was no evidence of a significant reduction in work and social functioning scores, no decline in specific COVID-19 concerns for family and friends, and no increase in help seeking over the 30-day study period. In addition, although the app was positively rated by most junior doctors, only a quarter of the participants completed the poststudy assessment, making it difficult to deduce what the remainder of the participants thought of the app.

Further assessment examining a possible relation between app use variables and depressive and anxiety symptoms showed effects in the proposed direction; however, these analyses had low power and did not yield any significant results. Similarly, increases in COVID-19 pandemic safety concerns could not be explained by app use. This, together with the lack of a control group, makes it impossible to conclude that the reductions in depressive and anxiety symptoms or increases in COVID-19 pandemic concerns were because of engagement with the app. However, it needs to be noted that dosage considerations based on classical intervention studies, such as medication trials, do not always apply to studies using self-directed eHealth interventions because participants in this study could choose their own “dosage” targets as a part of the app onboarding procedure [32]. The self-prescriptive nature of *Shift* was based on extensive user consultations with junior doctors to facilitate use and acceptance of the app [22]. As such, participants were free to decide how often and for how long they would use the app over the study period. It is, therefore, possible that junior doctors only used the app to feel better at a specific point in time, which may have elicited transient effects that did not materialize at the postassessment measurement. In the engagement literature, the concept of e-attainment proposes that the users of digital mental health tools engage with the technology for as long as they need to in order to reach their desired mental health goals [33]. Doctors may have only used the app if they deemed the contents to be useful for improving their mental health or used the app in conjunction with other strategies outside of the digital arena. With regard to personal safety concerns because of the COVID-19 pandemic, it is possible that the increased hospitalization rates during the second COVID-19 pandemic wave in 2020, which coincided with the data collection period of this study, were a more important predictor of safety considerations than any potential alleviation of concerns that the app contents could provide.

Among those who fully participated in this study, including the poststudy assessment, the app was generally well received, with most users rating the app highly or very highly. However, because of the extensive dropout rate, these results may be skewed, given that study completion was associated with more favorable app ratings and higher app use. This, in turn, implies that the app did not substantially engage a large portion of the study participants. Upon the inspection of app use variables, it appears that in particular, the help-seeking, psychoeducational, and value-based components of the app were underutilized and therefore require further user consultation and revision. The present sample expressed willingness to seek help for psychological problems and one-fifth of the baseline sample indicated having sought help from a mental health professional in the last 4 weeks, which could explain why this section of the app was not of great interest. However, given that the promotion of early help seeking for mental health concerns is one of the key aims of the app, the failure to engage users to familiarize themselves with help-seeking options needs to be addressed before a wider dissemination of the app may be considered. To do this successfully, a variety of barriers to help seeking should be overcome, some of which relate to pervasive mental health stigma in the medical profession and fears of being reported to medical regulators, and others relate to situational factors such as time constraints and the pandemic context [9,17,34,35].

This study has several strengths and limitations that affect the interpretability of the current findings. The strengths of this research are that *Shift* is the first app of its kind to address work and other common stressors of junior doctors, a cohort that has been known to be subjected to intensive work demands and declining mental health trajectories during trainee years. The app was created in close collaboration with junior doctors and was tested extensively at each development step to ensure that the tool is appropriate, useful, and does not incur harm. In addition, in response to the COVID-19 pandemic, we anticipated heightened stress in this group. Therefore, the study design was modified to allow all eligible candidates to receive the app immediately for download after registering for the study. We also minimized the number of reminder messages to only 1 message to avoid adding to participants’ stress by requesting their continued study participation. By avoiding these “push factors,” the study became more naturalistic and consequently had greater ecological validity than originally planned.

Limitations relate to the study design and attrition, which decrease the validity of this research. Owing to the novel pandemic context, we were unable to adopt established measures of COVID-19 pandemic safety concerns for doctors when we designed the study. The self-generated COVID-19 items as well as our adjustments to the General Help-Seeking Questionnaire were not pretested before their inclusion in this investigation. As such, it is possible that using these measures limited the validity of this research. The removal of a control group effectively changed the robust design to an extensive pilot evaluation. Because the recruitment phase coincided with the COVID-19 pandemic, historical effects are likely confounders to the observations presented in this paper. It also needs to be noted that a small proportion of the study participants showed moderate or severe symptoms of depression or anxiety. It is, therefore, possible that generally better-adjusted doctors who would not perceive study participation as an undue burden on them agreed to partake in this investigation. As such, it is possible that doctors who would potentially benefit the most from exposure to mental health information did not seek out this study, which impacts the generalizability of findings and may also tap into a greater issue of hard-to-reach persons who already present with mental health problems. In addition, the high attrition rate of this research may have further introduced selection bias, and the app feedback questions may have been subject to participants’ response bias. Although study completers did not vary in demographic characteristics and mental health profiles from those lost to follow-up, app acceptability and usability ratings, in particular, seem to have been skewed toward higher app use and more favorable app ratings among study completers. As for the high dropout rate, the analyses presented in this paper did not meet the initial expectations with regard to their achieved power. Therefore, it is possible that small but true effects may not have been detected. Future research should address these concerns and conduct a more robust examination of the effectiveness and overall usefulness of the app.

### Conclusions

Despite the crucial function that doctors play in ensuring the integrity and functioning of health care systems, doctors can feel undervalued and insecure in their ability to contribute what is needed to fulfill their role [36]. Considering the known barriers to addressing mental health concerns in this cohort, this study aimed to examine the usefulness of a tailored mental health app that can deliver initial mental health information and support options to junior doctors. Although the effectiveness of this approach is still in question, preliminary findings suggest that *Shift* seems to constitute a promising tool that may be able to reduce depressive and anxiety symptoms and improve outcomes for those who are willing to engage with digital mental health support. These results materialized despite a demanding pandemic context. Further research should seek to more robustly test the effectiveness for such apps on a wider scale among junior doctors. Although these types of individual interventions should constitute a part of the response to support physicians’ mental health, they need to be a part of a broader suite of interventions that also consider the systemic and organizational factors that impact the health and well-being of health care workers.

## Appendix

Multimedia Appendix 1 Overview and categorization of *Shift* app activities.

## Abbreviations

CBT: cognitive behavioral therapy
EMM: estimated marginal mean

## Data Availability

Data from all participants were included in this paper, provided participants did not withdraw their informed consent to participate. We did not seek permission from participants to make deidentified data available as per the open science framework. Consequently, data are not publicly available because of ethical restrictions.
