# Correction: Preliminary Investigation of Shift, a Novel Smartphone App to Support Junior Doctors’ Mental Health and Well-being: Examination of Symptom Progression, Usability, and Acceptability After 1 Month of Use

**DOI:** 10.2196/47277

**Published:** 2023-04-03

**Authors:** Samineh Sanatkar, Isabelle Counson, Andrew Mackinnon, Alexandra Bartholomew, Nick Glozier, Samuel Harvey

**Affiliations:** 1 Black Dog Institute Randwick Australia; 2 School of Psychiatry, Faculty of Medicine, University of New South Wales Sydney Kensington Australia; 3 Central Clinical School, Faculty of Medicine and Health, University of Sydney Camperdown Australia

In “Preliminary Investigation of Shift, a Novel Smartphone App to Support Junior Doctors’ Mental Health and Well-being: Examination of Symptom Progression, Usability, and Acceptability After 1 Month of Use” (J Med Internet Res 2022;24(9):e38497) the authors noted one syntax error:

A total of 222 female (n=156, 70.3%; mean age 29.2, SD 4.61 years) junior doctors provided full baseline data.

The corrected version is:

A total of 222 (n=156 female, 70.3%; mean age 29.2, SD 4.61 years) junior doctors provided full baseline data.”

The correction will appear in the online version of the paper on the JMIR Publications website, together with the publication of this correction notice on April 3, 2023. Because this was made after submission to PubMed, PubMed Central, and other full-text repositories, the corrected article has also been resubmitted to those repositories.

