# Supplementary material for: Preliminary Investigation of Shift, a Novel Smartphone App to Support Junior Doctors’ Mental Health and Well-being: Examination of Symptom Progression, Usability, and Acceptability After 1 Month of Use
Source: J Med Internet Res. 2022 Sep 21;24(9):e38497. doi: 10.2196/38497 (PMC9536518; doi:10.2196/38497)
Supplement: Multimedia Appendix 1 [file jmir_v24i9e38497_app1.docx]

# Appendix

*Supplementary Material Table 1*

*Overview and Categorisation of Shift App Activities*

| App Category | App Sub-Category | App Activity Name | Internal Grouping |
| --- | --- | --- | --- |
| Mental Health | Evaluating Thoughts |  | Cognitive Behavioural |
|  |  | Introduction to Unhelpful Thoughts |  |
|  |  | Unhelpful Thoughts |  |
|  |  | Cognitive Biases |  |
|  |  | Introduction to Evaluating Thoughts |  |
|  |  | Evaluating Thoughts |  |
|  |  | Worry Decision Tree |  |
|  |  | Cognitive Therapy Review |  |
|  | Taking Action |  | Values |
|  |  | Your Values |  |
|  |  | Relax, Refocus, Review |  |
|  |  | Meaningful Actions 1 - 5 |  |
|  | Common Concerns |  | Psychoeducation |
|  |  | Depression |  |
|  |  | Anxiety |  |
|  |  | Health Anxiety |  |
|  |  | Burnout |  |
|  |  | Post-Traumatic Stress |  |
|  |  | Alcohol and Drugs |  |
|  |  | Wellbeing Plan |  |
| Seeking Help |  |  | Get Help |
|  |  | Get Help Now |  |
|  |  | Help at Work |  |
|  |  | Help at Home |  |
|  |  | Mandatory Reporting |  |
| Lifestyle | Mindfulness |  | Mindfulness |
|  |  | Introduction to Mindfulness |  |
|  |  | Seeing the Horizon |  |
|  |  | Grounding Anchor |  |
|  |  | Calming Breath |  |
|  |  | Loving Kindness |  |
|  |  | Cargo Thoughts |  |
|  |  | Breathing Wind |  |
|  |  | Lapping Ocean |  |
|  |  | Morning Bell |  |
|  |  | Progressive Muscle Relaxation |  |
|  |  | Gratitude |  |
|  | Sleep and Fatigue |  | Work and Lifestyle |
|  |  | Sleep Health |  |
|  |  | Adjust to *Shift* Work |  |
|  |  | Work-Life Balance |  |
|  | Diet and Exercise |  |  |
|  |  | Diet |  |
|  |  | Exercise |  |
|  |  | Get Active |  |
|  | Relationships |  |  |
|  |  | Social Support |  |
|  |  | Help a Friend |  |
| Work |  |  |  |
|  |  | Exams and Interviews |  |
|  |  | Rural/Regional Placements |  |
|  |  | Bullying |  |
|  |  | Grief |  |
|  |  | Calling for a Consult |  |
|  |  | Feeling Inadequate |  |
| Dealing with a Pandemic* |  |  |  |

*Note*. *This additional module was affixed to the home screen in response to the coronavirus pandemic
